# Winter coexistence in herbivorous waterbirds: Niche differentiation in a floodplain, Poyang Lake, China

**DOI:** 10.1002/ece3.8314

**Published:** 2021-11-15

**Authors:** Junpeng Bai, Huan Zhang, Hongkang Zhou, Shu Li, Bin Gao, Peng Chen, Long Ma, Zhifeng Xu, Zhen Zhang, Changxin Xu, Luzhang Ruan, Gang Ge

**Affiliations:** ^1^ School of Life Sciences Key Laboratory of Poyang Lake Environment and Resource Utilization Ministry of Education Nanchang University Nanchang China; ^2^ Ministry of Ecology and Environment Nanjing Institute of Environmental Sciences Nanjing China; ^3^ Institute of Life Science and School of Life Sciences Jiangxi Province Key Laboratory of Watershed Ecosystem Change and Biodiversity Center for Watershed Ecology Nanchang University Nanchang China

**Keywords:** conservation measures, endangered species, food abundance, foraging habitats, hydrological fluctuations

## Abstract

The classical niche theory supports the idea that stable coexistence requires ecological differences between closely related species. However, information on waterbirds coexistence in the entirely landlocked freshwater system of Poyang Lake is not well understood, especially when the available biomass of their food in the area decreases. In this study, we tested the ecological segregation mechanisms in the 2015/2016 and 2016/2017 wintering periods among eight herbivorous waterbirds (including the Siberian crane *Grus leucogeranus*, hooded crane *Grus monacha*, white‐naped crane *Grus vipio*, common crane *Grus grus*, greater white‐fronted goose *Anser albifrons*, bean goose *Anser fabalis*, swan goose *Anser cygnoides*, and tundra swan *Cygnus columbianus*) at Poyang Lake. Using field observations and species niche and foraging habitat selection models, we investigated the abundance, distribution, and food sources of these eight waterbird species to quantify and compare their habitat use and ecological niches. Our results showed that niche segregation among the waterbirds, with respect to food types, time, and spatial location, allow them to coexist and use similar resources. The water level gradually receded in the sub‐lakes of the Poyang Lake, which could provide food sources and various habitats for wintering herbivorous waterbirds to coexist. We demonstrated that the differences in habitat use could mitigate interspecific competition, which may explain the mechanism whereby waterbirds of Poyang Lake coexist during the wintering period, despite considerable overlap in the dietary niches of herbivorous waterbirds.

## Graphical abstract

Niche separations among waterbirds with respect to food types, time, and spatial locations facilitate coexistence. Water levels in sub‐lakes strongly affect the food sources and habitat availability, affecting abundance and dispersal of waterbirds. Differences in habitat use amongst waterbirds may mitigate interspecific competition, avoiding exclusion, in winter at Poyang Lake.

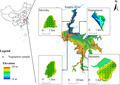

## 1 INTRODUCTION

Wetlands are critical foraging areas for waterbird species, and these areas sustain a high level of biodiversity. It is often believed that their relatively high productivity is the principal factor that determines the coexistence of sympatric species using similar food resources during breeding and non‐breeding seasons (Chatterjee et al., 2020; Wiens, 1989). Based on the classical niche theory, stable coexistence of species requires segregation of their niches to mitigate competition for limited food resources, thereby allowing multiple species to forage simultaneously within the same region (MacArthur, 1958; Pianka, 1974). However, the complex dynamics of wetland structures in response to hydrological regime shifts or climate change could limit the food sources for waterbirds, thereby enhancing dietary competition between species (Cumming et al., 2012; Lorenzon et al., 2017). Specifically, many waterbird species show large overlaps in their diets and foraging sites (Henry & Cumming, 2017).

Habitat use is another important niche dimension for the coexistence of waterbird species, and the overlap of their niches can be reduced through resource partitioning and habitat differentiation (Schoener, 1974; Xu et al., 2021). The habitat availability of waterbirds in seasonally inundated wetlands is strongly affected by water level changes (Baschuk et al., 2012; Holm & Clausen, 2006). As habitats vary in structures and resources seasonally, waterbird species with different feeding guilds migrate to favorable areas. The relationship between water level and habitat availability strongly affects the waterbird species that occupy ecological niches at different water depths (Mei et al., 2016; Polla et al., 2018). For example, geese generally forage in exposed riparian grasslands, whereas cranes favorably forage in mudflats or shallow water areas (Jia et al., 2019). Tundra swans prefer to feed in deeper water, and diving birds forage at high water depths (Jiang et al., 2015). When food resources are scarce, waterbirds adapt various strategies for coexistence that enable them to optimally utilize the available food resources. Field evidence showed that Siberian cranes (*Grus leucogeranus*) shifted their diet from tubers of submerged macrophyte to a different plant (*Potentilla limprichtii*) when their preferred tuber was extremely scarce (Jia et al., 2013). Many waterbird species have evolved unique anatomical specializations, such as special beak shapes, long necks and tarsi, and behavioral specializations that enable them to forage in different areas with variable water depths (Elphick, 2008; Ntiamoa‐Baidu et al., 1998).

Poyang Lake is a large floodplain wetland and an important Ramsar site, to which hundreds of thousands of wintering waterbirds migrate every year, particularly herbivorous waterbird species, such as geese, cranes, and swans (Barter et al., 2005; Ji et al., 2007; Ruan et al., 2018). Poyang Lake provides various habitats and abundant food resources for the waterbirds to coexist while foraging and wintering as the water level recede gradually during the low water period (Aharon‐Rotman et al., 2017; Yang et al., 2020). As a result, this wetland provides an excellent opportunity to examine the coexistence of herbivorous wintering waterbirds that use different foraging habitats in response to lake water level. In this study, we explored the potential mechanism through which herbivorous waterbirds coexist for wintering at Poyang Lake in order to develop appropriate protection measures. Moreover, the following two hypotheses were tested: (1) niche partitioning occurs among overwintering waterbirds for food, space, and time, which facilitates coexistence and (2) gradual falling water levels in dry season may affect the availability of food and suitable habitat area, affecting waterbirds’ abundance and distribution at Poyang Lake.

## 2 MATERIAL AND METHODS

### 2.1 Study area

**FIGURE 1:**
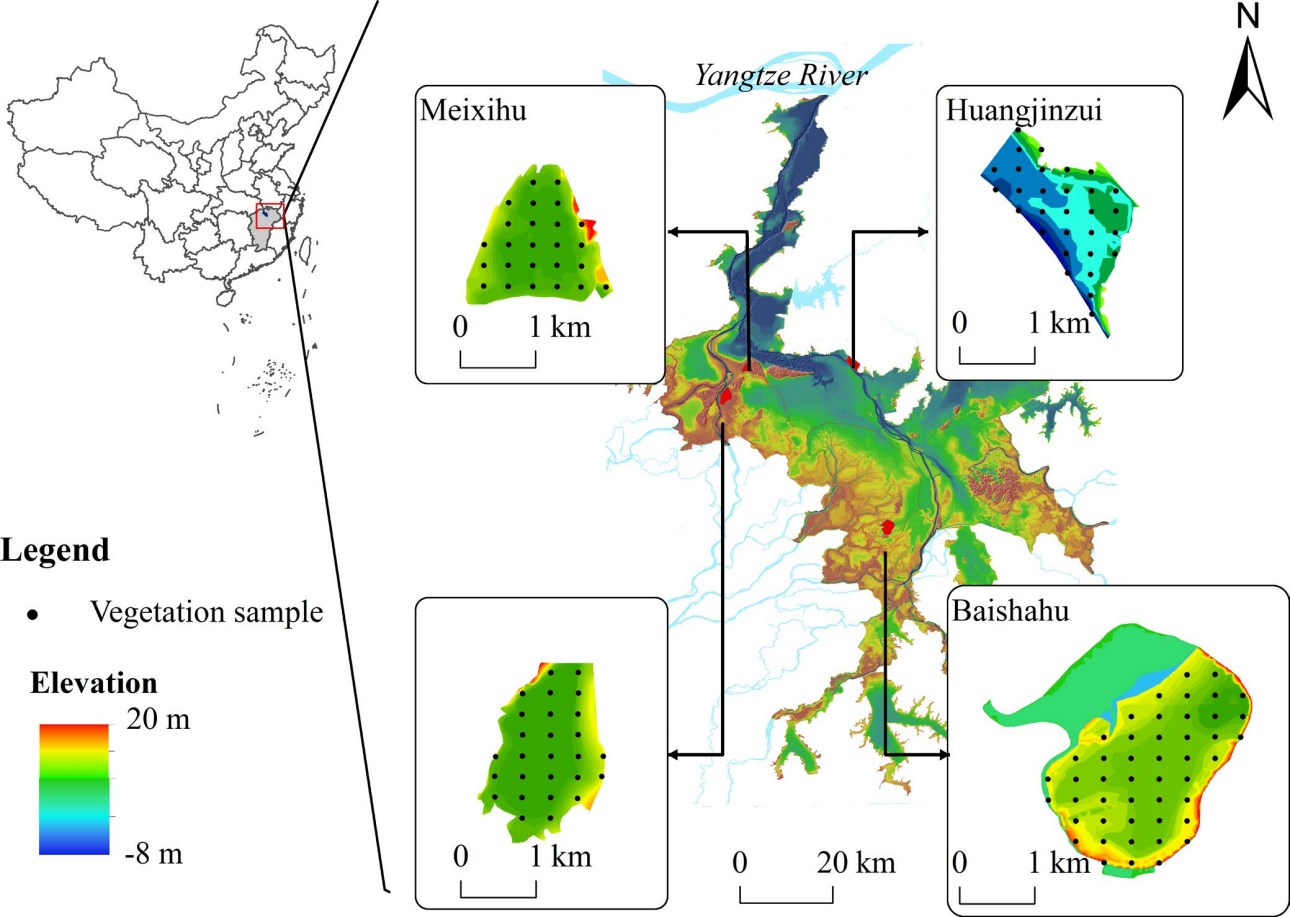
Location of four study areas (Meixihu, Changhuchi, Baishahu, and Huangjinzui) in Poyang Lake, China. Black dots represent the sampling sites of vegetation survey

Poyang Lake (28°11ʹ–29°51ʹN, 115°49ʹ–116°46ʹE) is the largest freshwater lake in China, and it is situated in the middle reaches of the Yangtze River and the northern part of Jiangxi Province (Figure 1). This lake is one of two large lakes that are freely connected to the Yangtze River. Owing to its monsoonal climate, Poyang Lake exhibits considerable seasonal and interannual variation in water levels (Min, 2007; Zhang et al., 2014).

The considerable differences in water levels shape the distinct landscapes between the wet summer and dry winter seasons in Poyang Lake. During the wet period, landscape in the wetland mainly consists of open water, with the water surface area reaching 4000 km^2^. However, besides permanent open water, the wetland landscape mainly consists of herbaceous meadows, mudflats, and isolated water bodies during the dry period. It is a favorable habitat for migratory waterbirds overwintering in this period (Barter et al., 2005; Ji et al., 2007).

This study was conducted at three sub‐lakes, namely Meixihu (116°03′27″E, 29°13′09″N), Changhuchi (115°59′22″E, 29°08′09″N), and Baishahu (116°19′50″E, 28°54′41″N), and an area of Poyang Lake shoreline, Huangjinzui (116°16′05″E, 29°13′41″N) (Figure 1). These three sub‐lakes are seasonally inundated waterbodies that are controlled by sluices (Chen & Zuo, 2001; Xia et al., 2016). Huangjinzui lies at the Duchang migratory bird reserve and is adjacent to the open water of the Poyang Lake. The water level in this area dramatically decreases, and large herbaceous meadows form as water recedes during the dry season.

During the low water period, the three sub‐lakes consist of water bodies, mudflats, and vast meadows with herbaceous vegetation that are mainly occupied by *Carex* spp. and sedge communities (Jian et al., 2001; Zhang, Li, et al., 2012; Zhang, Yin, et al., 2012). The inundated areas are often covered with submerged vegetation and are dominated by *Vallisneria natans*, *Hydrilla verticillata*, and *Ceratophyllum demersum*, which could provide plenty of food resources for wintering waterbirds. During the low water period, Huangjinzui dries up, and hence, this study area was dominated by herbaceous meadows, for example, *Polygonum criopolitanum*, *Carex* spp. or other grasses.

### 2.2 Data collection

#### 2.2.1 Water level data collection

The water levels in the study sites were monitored using the nearest hydrographic stations during the flood period. As the water bodies were isolated from open water during the dry period, we monitored the water levels of the three sub‐lakes at the sluices (Yellow Sea Datum) at the beginning and middle of each month. Additionally, the water level of Huangjinzui was recorded by the Duchang hydrological station throughout the year (Figure S1).

#### 2.2.2 Vegetation surveys

Considering the overwintering period of the waterbirds at Poyang Lake, vegetation surveys were conducted in early October and late March of 2015/2016 and 2016/2017. Overall, 130 vegetation sampling points were investigated each time, including 25 from Meixihu, 27 from Changhuchi, 48 from Baishahu, and 30 from Huangjinzui. During sampling, a hand‐held global positioning system (GPS) (Garmin eTrex 6, Taiwan, China) was used to determine the geographic coordinates of the sampling locations.

We collected tuber samples from the submerged vegetation using a stainless steel mud collector. Five replicate samples were randomly collected near each sampling point and brought back to the laboratory. Tuber samples were carefully washed with water and weighed using electronic scales (accuracy 0.001 g). These samples were then placed in an oven for 30 min at 105℃ and then dried at 55℃ for 2 days to obtain the dry weights. The biomass of the submerged vegetation tubers was calculated for both dry and fresh weights.

#### 2.2.3 Waterbird surveys

Waterbird data were obtained from surveys conducted at the study sites between 2015/2016 and 2016/2017 over two overwintering periods. Generally, migratory waterbirds winter at Poyang Lake from October to March of the following year. Therefore, we surveyed the waterbirds at the study sites twice per month from early October to late March and recorded their species and abundance as well as their foraging sites in each 300 × 300 m quadrat (*N* = 120) comprising the vegetation sampling point on a printed paper map of the area. We did not conduct the surveys on days with extreme weather (i.e., foggy, windy, and heavy rain days) to avoid biased measurements. Before counting, the observation of foraging behavior was typically conducted for 10–20 min. Surveys were conducted simultaneously at fixed locations along fixed routes to avoid repeated counting and to improve counting accuracy. In order to ensure that the monitoring field covered the entire area and did not interfere with the behavior of the waterbirds, one or two permanent observation locations (fixed points) were established at each sub‐lake. We adopted the look—see counting method (Barter et al., 2005; Cao et al., 2008), and waterbird species were distinguished based on the methods suggested by Mackinnon and Phillipps (2000) and Barter et al. (2005).

### 2.3 Habitat variables

We collected data related to geographical characteristics, habitat features, and human disturbance factors for each studied site (Table S1). The elevation data were provided by the Department of Water Resources, Jiangxi Province (Yellow Sea Datum). The geographic locations of the vegetation quadrats were determined using a GPS device. Given the difficulty to accurately identify waterbirds’ locations in the field. We recorded species identity and their numbers referring to the grid on a map with some obvious references, such as micro‐topology, vegetation. As a result, location data of waterbirds in this study were relatively accurate. The World Geodetic System 1984 was used while recording all the locations. The difference between elevation and water level was represented as the water table (WT). The biomass of the submerged macrophyte tubers before overwintering (TBI) is a potential food for tuber eaters, whereas the biomass after overwintering (TBII) is unused food. The availability of food (TBD) is the difference between tuber biomass before and after overwintering. The height (CHC) and coverage (CCD) of *Carex* spp. before overwintering are represented as CHI (cm) and CCI (%), respectively, and those after overwintering are represented as and CHII (cm) and CCII (%), respectively. The height and coverage of *Carex* spp. before and after overwintering can be used to develop indices of food availability for short grass foragers. We also measured the distance (m) to the nearest road (DR), village (DV), and lake center (DC) from each foraging location of the waterbirds. All distances were calculated using ArcGIS (version 10.1).

All data were imported into the ArcGIS software, including the latitudes and longitudes of the waterbird foraging positions, geographical characteristics, habitat, food, and hydrological and human disturbance factors. All foraging habitat characteristics are listed in Table S1. Several 300 × 300 m quadrats (*N* = 120) comprising vegetation sampling points were interpolated using the inverse distance weighted method and the number of species investigated in each quadrat was counted separately (Bancroft et al., 2002). Subsequently, the factors influencing the feeding habitats of waterbirds were extracted according to their feeding sites in each quadrat. Each dataset included 15 habitat factors for each quadrat in 2 years (Table S1). Thus, each quadrat had 2880 observations (24 times × 120 quadrats, including quadrats with zero).

### 2.4 Statistical methods

The values are reported as mean ± SD. The normality of the distributions was assessed using the Kolmogorov–Smirnov test, with the significance level set at 0.05 (Lilliefors, 1967). When the data were not normally distributed (Table S2), Spearman's rank correlation test was performed to assess the correlation among the habitat parameters (Table S3; Maritz, 1995). One‐way analysis of variance and Duncan's new multiple range test (Hsu, 1996) were used to evaluate the differences between the foraging habitat characteristics of eight species. We focused only on the dominant species of tuber eaters and *Carex* spp. foragers among the wintering herbivorous waterbirds in the Poyang Lake area, namely, the Siberian crane *Grus leucogeranus*, hooded crane *Grus monacha*, white‐naped crane *Grus vipio*, common crane *Grus grus*, greater white‐fronted goose *Anser albifrons*, bean goose *Anser fabalis*, swan goose *Anser cygnoides*, and tundra swan *Cygnus columbianus* (Barzen et al., 2009; Wang, Fox, et al., 2013; Wang, Jia, et al., 2013).

Principal component analysis was used to analyze the variation within and among these variables for different bird species (Conner & Adkisson, 1977). We selected the first (PC1), second (PC2), and third (PC3) principal components whose eigenvalues were greater than 1 and whose contribution values were greater than 10.00%. We employed R package nicheROVER (Swanson et al., 2015) to calculate the pairwise of niche overlap probability for eight waterbird species and niche width of each bird in three dimensions (PC1, PC2, and PC3). We calculated corresponding items using the equations below.

1. Niche overlap:

$$O\left(\begin{array}{c} A\\ B \end{array}\right)=\mathrm{Pr}\left(X_{A}\epsilon N_{R}\left(B\right)\right)$$

where *X_A_* and *X_B_* was correspond to randomly selected principal components in three dimensions from different bird species, and let *N_R_* (*A*) and *N_R_* (*B*) denote their respective niche regions. W. There was a probability of an individual from bird species *A* to be found in the modeled niche region of bird species *B* and vice versa (i.e., overlap probability). The difference between these two values facilitates the assessment of asymmetric niche overlap between groups. We modeled 1000 samples and ran 1000 iterations to compute niche regions with a probability level of alpha 0.95 (i.e., 95.00% probability) and 95.00% confidence intervals, and used 10 Monte Carlo draws to plot the elliptical projection (i.e., default priors).

- Niche width:

$$N_{s}=\int x\epsilon N_{R}dx$$

where *N_R_* was a given niche region in three dimensions (PC1, PC2, and PC3), and the niche width is defined as the hypervolume of this region and calculated the size of an elliptical niche region for eight waterbird species.

Due to little difference in the mean numbers of wintering waterbirds between 2015/2016 and 2016/2017, we merged the data from the 2 years into a single data pool. We employed generalized linear models with Poisson family distribution and a log‐link function to predict foraging habitat selection with the number of waterbirds as the dependent variable (Zuur et al., 2009). To reduce over fitting caused by redundant variables, we selected the most explanatory and uncorrelated variables and eliminated the others based on the highest correlation coefficients (Spearman's rank correlation, |*r*| > 0.60; Soh et al., 2002; Inselman et al., 2015). Thereafter, the variables that provided the most meaningful biological interpretation were retained, and the others were eliminated from further analyses, resulting in a set of eight variables (TBD, CHC, CCD, WL, WT, DR, DV, and DC) as the fixed variables. We used a backward selection procedure to eliminate the terms gradually based on their decreasing *p*‐values. Model selection was based on Akaike information criterion corrected (AICc) for a small sample size (Burnham & Anderson, 2004). Models with ΔAICc < 2 have support for being the best model in a set. Additionally, we used Akaike weights (*ω_i_*) as an indicator to support the best models.

All statistical analyses were performed using the software R 3.6.1 (R Core Team, 2019).

## 3 RESULTS

### 3.1 Abundance and distribution of herbivorous waterbirds

**FIGURE 2:**
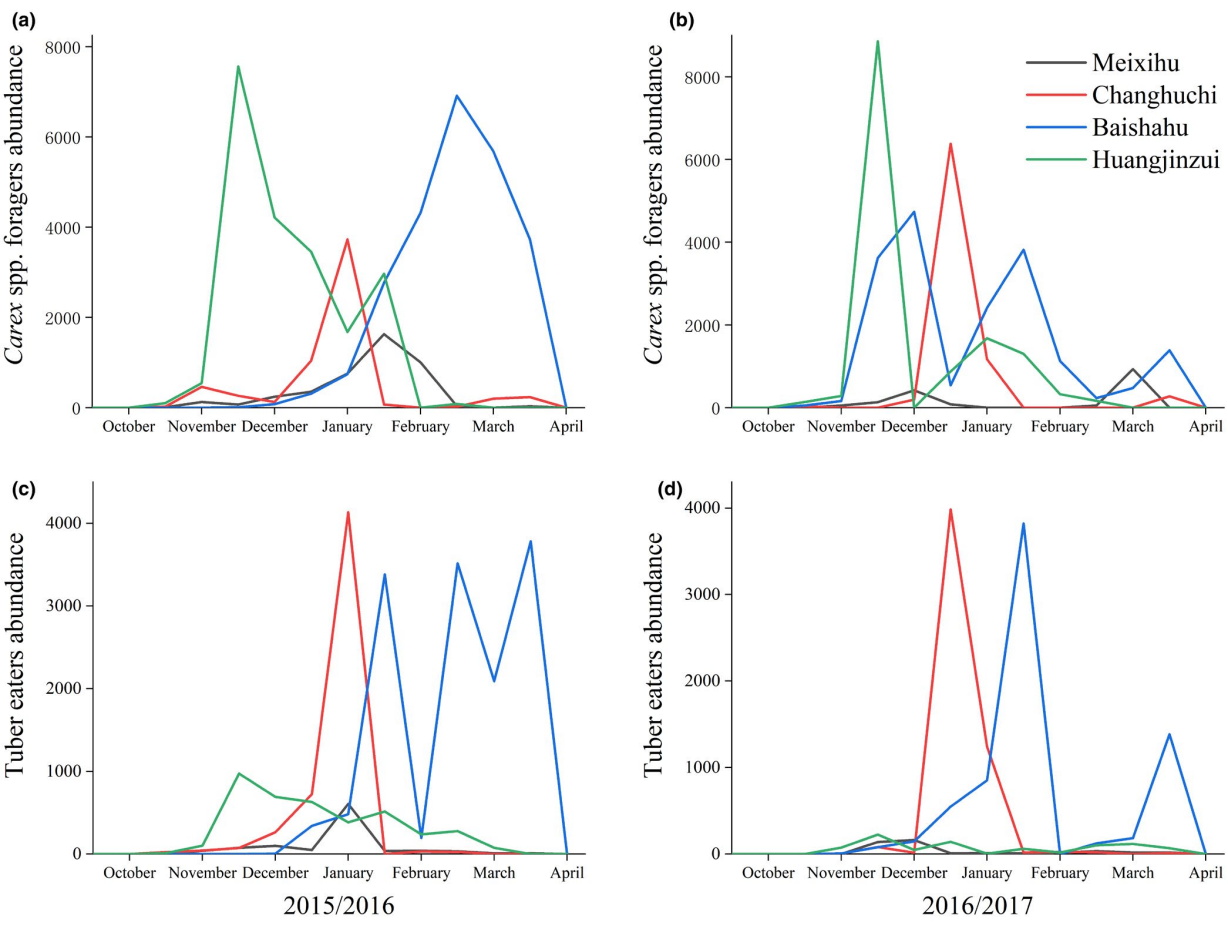
The abundance of *Carex* spp. foragers and tuber eaters at four study areas of Poyang Lake. (a): the abundance of *Carex* spp. foragers in 2015/2016, (b): the abundance of tuber eaters in 2015/2016, (c) the abundance of *Carex* spp. foragers in 2016/2017, (b): the abundance of tuber eaters in 2016/2017

The number of herbivorous waterbirds in the four study sites varied throughout the survey period (Figure 2 and Table S4). For the *Carex* spp. foragers, the number of individuals peaked at Huangjinzui in mid‐November in 2015/2016 and 2016/2017, but peaked in early January at three sub‐lakes. Moreover, a small peak in number of waterbirds occurred at the three sub‐lakes around February. For tuber eaters, the overall trend was similar to that of the *Carex* spp. foragers, but more individuals were found at the three sub‐lakes than at Huangjinzui.

**FIGURE 3:**
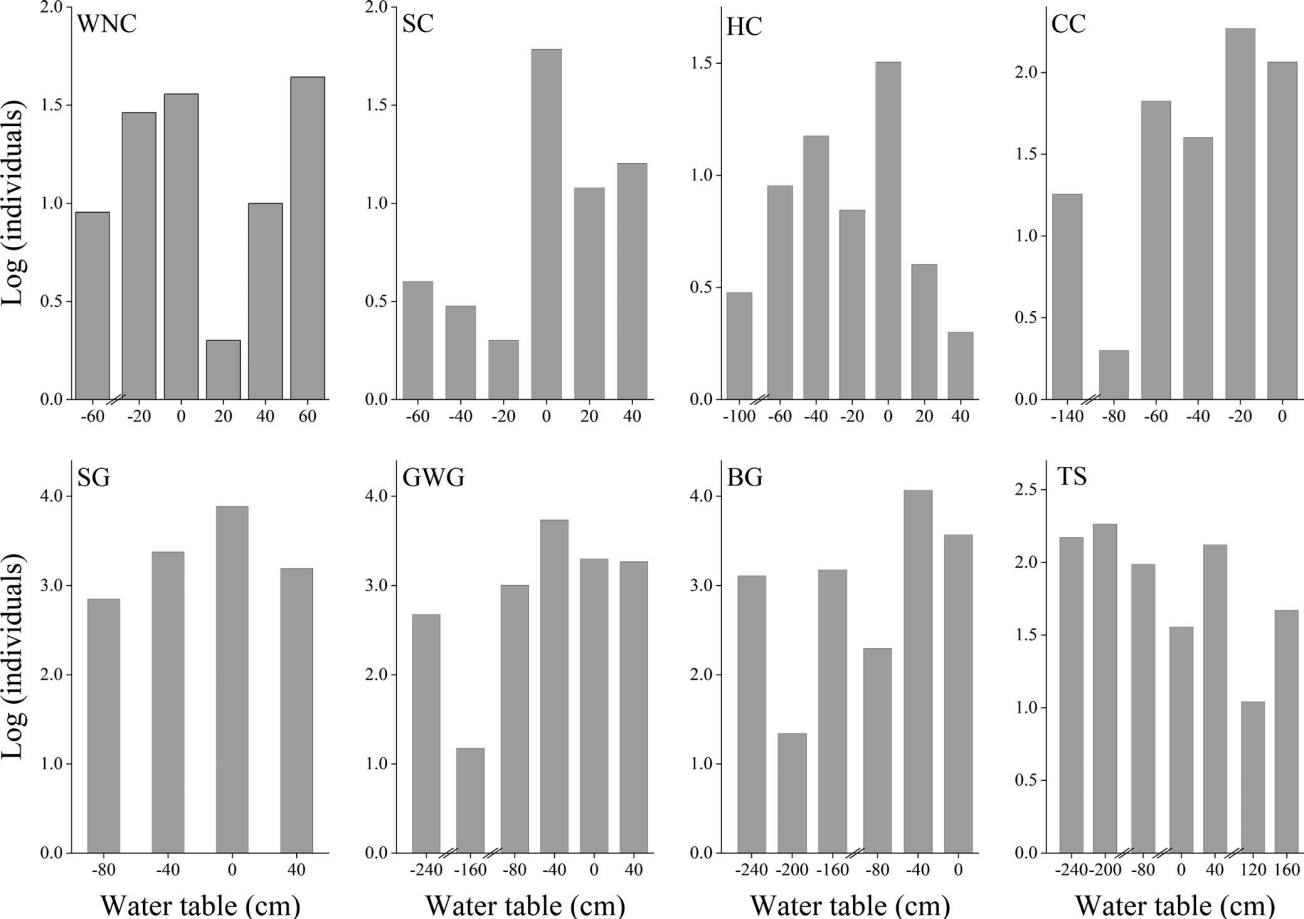
Distribution of individuals of eight waterbird species according to the water table. BG, bean goose; CC, common crane; GWG, greater white‐fronted goose; HC, hooded crane; SC, Siberian crane; SG, swan goose; TS, tundra swan; WNC, white‐naped crane

The results showed that the movement range of the tuber eaters was relatively concentrated (WT = −140.0–58.9 cm), except for the tundra swan (WT = −236.0–178.0 cm), whereas that of the *Carex* spp. foragers were wider (WT = −246–42 cm). The eight species of waterbirds were densely distributed along with the WT (Figure 3). The distribution of the white‐naped crane ranged from a WT of −60 cm to 60 cm, whereas for the Siberian crane the distribution ranged from a WT of −60 cm to 40 cm. Although the hooded cranes were distributed in a similar WT region as the Siberian cranes, the hooded cranes waded shallower than the Siberian cranes. In addition, the common cranes were distributed in the WT region ranging from −140 cm to 0 cm and were mainly concentrated at −20 cm. Goose species distributed in the WT region ranged from −240 cm to 40 cm. The distribution of the tundra swans was wider than that of other herbivores.

### 3.2 Habitat characteristics

**FIGURE 4:**
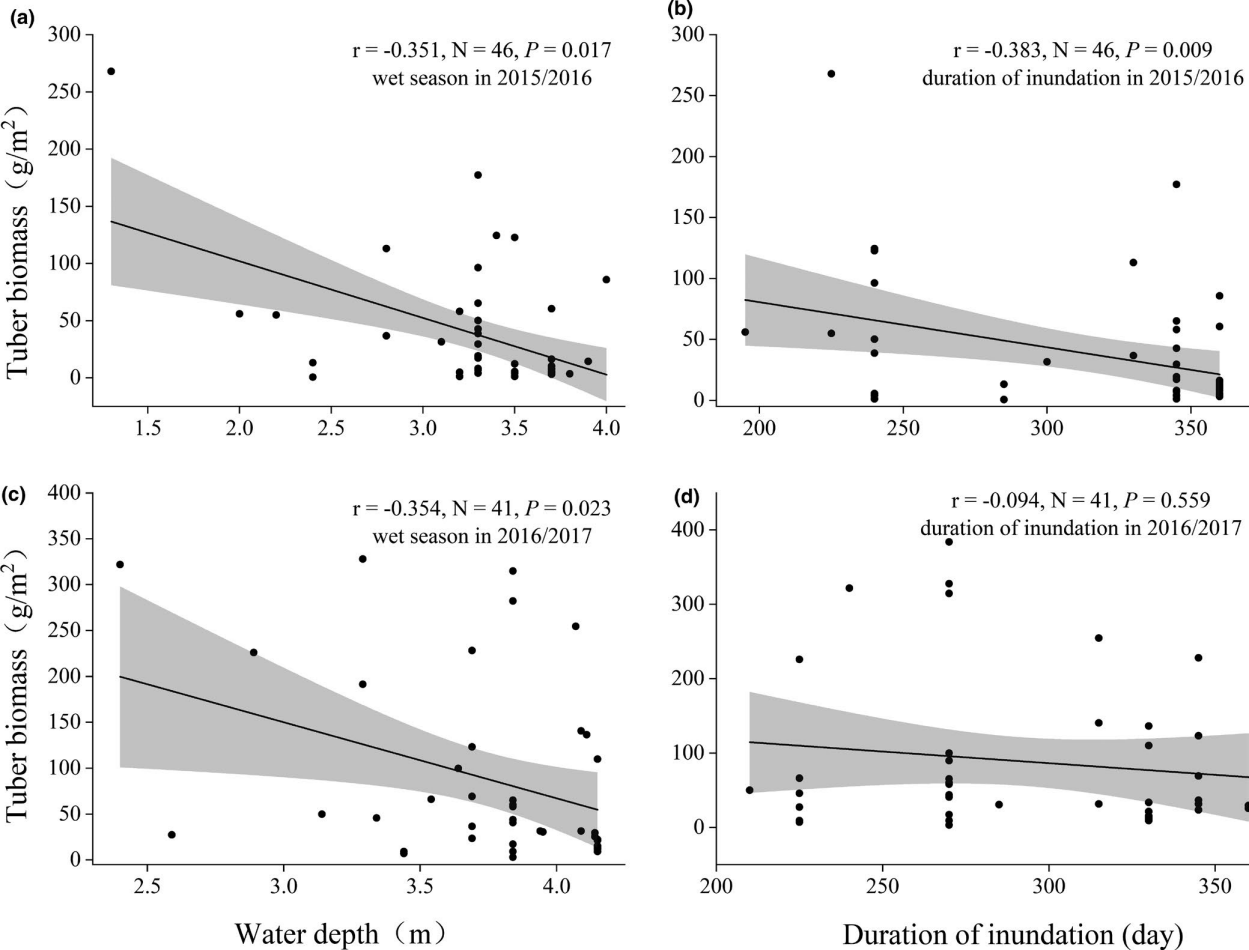
The relationship between water depth (a, c), duration of inundation (b, d) and tuber biomass of *Vallisneria* spp. (a): the relationship between tuber biomass and water depth in 2015/2016, the relationship between tuber biomass and duration of inundation in 2015/2016, (c): the relationship between tuber biomass and water depth in 2016/2017, (d): the relationship between tuber biomass and duration of inundation in 2016/2017

The potential food resources for the waterbirds were distributed in a step‐like manner with peaks in the study sites, and their distribution was spatially uneven (Figure S2). In general, *Carex* spp. was distributed in the higher elevation areas. In contrast, the tubers of *Vallisneria* spp. were mainly distributed in the littoral zone or mudflats in the lower elevation areas. Linear regression analysis revealed a significant negative correlation of sub‐lake water depth with tuber biomass during the wet season in 2015/2016 (*r* = −0.351, *N* = 46, *p* = .017) and 2016/2017 (*r* = −.354, *N* = 41, *p* = .023; Figure 4).

Our results revealed substantially differences among the favorable habitat characteristics of the eight waterbird species (Table 1). With regard to food source factors, the TBI, TBII, and TBD were the highest in habitats used by tundra swans, whereas the lowest values were obtained in habitats used by the greater white‐fronted geese and bean geese. The height and coverage of *Carex* spp. used by bean geese had the highest values of CHI, CHII, CCI, and CCII, whereas the lowest values of CHI and CHII were found in the habitats of the tundra swan and those of CCI and CCII in the habitats of the Siberian crane. After the overwintering period, the coverage of *Carex* spp. was significantly higher than other vegetation species in the habitat of swan geese, whereas the lowest CCD value was estimated for tundra swans’ habitat.

**TABLE 1:** Variables of different habitat characteristics of eight waterbird species and the ANOVA and Duncan's new multiple range test

	SC	HC	WNC	CC	GWG	BG	SG	TS
TBⅠ (g/m^2^)	50.40 ± 49.17^a^	45.42 ± 41.89^a^	57.91 ± 56.52^a^	45.75 ± 50.40^a^	21.65 ± 33.69^a^	35.93 ± 72.00^a^	40.57 ± 80.13^a^	171.04 ± 134.88^b^
TBⅡ (g/m^2^)	14.33 ± 20.49^a^	9.58 ± 8.48^a^	6.11 ± 10.65^a^	5.57 ± 8.63^a^	4.04 ± 6.19^a^	18.83 ± 58.34^a^	19.20 ± 71.07^a^	114.75 ± 116.20^b^
TBD (g/m^2^)	36.07 ± 35.44^abc^	35.84 ± 37.23^abc^	51.80 ± 50.46^bc^	40.18 ± 45.08^abc^	17.61 ± 31.69^a^	17.09 ± 25.93^a^	21.37 ± 27.96^ab^	56.29 ± 71.14^c^
CHⅠ (cm)	3.83 ± 5.41^ab^	4.82 ± 5.81^abc^	3.47 ± 5.15^ab^	7.18 ± 5.28^bc^	4.20 ± 3.76^ab^	8.62 ± 4.82^c^	7.12 ± 5.31^bc^	2.83 ± 3.12^a^
CHⅡ (cm)	3.96 ± 5.59^ab^	4.95 ± 5.92^abc^	3.62 ± 5.29^ab^	7.44 ± 5.38^bc^	4.46 ± 4.06^ab^	8.97 ± 4.85^c^	7.31 ± 5.48^bc^	3.03 ± 3.27^a^
CHC (cm)	0.13 ± 0.22^a^	0.13 ± 0.26^a^	0.14 ± 0.22^a^	0.26 ± 0.22^a^	0.27 ± 0.47^a^	0.35 ± 0.27^a^	0.19 ± 0.46^a^	0.20 ± 0.20^a^
CCⅠ (%)	0.18 ± 0.26^a^	0.26 ± 0.32^abc^	0.22 ± 0.36^ab^	0.49 ± 0.32^cd^	0.32 ± 0.28^abcd^	0.53 ± 0.28^d^	0.45 ± 0.31^bcd^	0.19 ± 0.22^a^
CCⅡ (%)	0.16 ± 0.24^a^	0.24 ± 0.31^abc^	0.21 ± 0.34^ab^	0.46 ± 0.31^cd^	0.29 ± 0.27^abcd^	0.51 ± 0.27^d^	0.42 ± 0.30^bcd^	0.18 ± 0.22^a^
CCD (%)	0.02 ± 0.03^abc^	0.02 ± 0.02^abc^	0.01 ± 0.02^ab^	0.03 ± 0.03^c^	0.03 ± 0.03^bc^	0.02 ± 0.02^abc^	0.03 ± 0.03^c^	0.00 ± 0.01^a^
Elev (m)	13.08 ± 0.65^cd^	13.15 ± 0.60^d^	12.52 ± 1.67^bcd^	12.81 ± 0.77^bcd^	12.33 ± 0.79^bc^	12.19 ± 1.16^b^	12.49 ± 0.84^bcd^	10.59 ± 0.17^a^
WL (m)	13.00 ± 0.46^d^	13.01 ± 0.48^d^	12.37 ± 0.74^cd^	12.60 ± 0.75^cd^	11.88 ± 1.49^bc^	11.39 ± 1.95^b^	12.22 ± 0.92^bcd^	9.89 ± 1.51^a^
WT (cm)	−8.10 ± 48.18^b^	−14.28 ± 32.61^b^	−15.76 ± 126.19^b^	−21.35 ± 30.71^ab^	−44.79 ± 77.48^ab^	−79.30 ± 88.54^a^	‐27.27 ± 31.66^ab^	‐69.44 ± 156.50^ab^
DR (m)	734.08 ± 267.62^a^	744.76 ± 319.91^ab^	999.56 ± 343.73^b^	825.16 ± 387.42^ab^	648.61 ± 302.33^a^	597.28 ± 272.62^a^	713.33 ± 359.30^a^	610.11 ± 229.08^a^
DV (m)	1077.09 ± 528.03^bc^	910.93 ± 488.88^ab^	1149.59 ± 318.99^bc^	1424.69 ± 444.75^c^	1258.63 ± 671.57^bc^	1034.33 ± 557.88^abc^	1229.12 ± 567.24^bc^	643.00 ± 186.58^a^
DC (m)	601.73 ± 261.55^a^	563.73 ± 227.95^a^	662.24 ± 245.11^a^	578.29 ± 199.95^a^	730.00 ± 264.57^a^	689.62 ± 196.31^a^	681.97 ± 274.40^a^	748.87 ± 199.64^a^

Note

Significant difference at the 0.05 level. a, b, c and d represent different groups respectively, and there is significant difference between a, b, c and d groups.

Abbreviations: BG, bean goose; CC, common crane; CCⅠ, *Carex* spp. Coverage Ⅰ; CCⅡ, *Carex* spp. Coverage Ⅱ; CCD, Carex spp. coverage decrease; CHⅠ, *Carex* spp. Height Ⅰ; CHⅡ, *Carex* spp. height Ⅱ; CHC, Carex spp. height changes; DC, Distance from center; DR, Distance from road; DV, Distance from village; Elev, Elevation; GWG, greater white‐fronted goose; HC, hooded crane; SC, Siberian crane; SG, swan goose; TBⅠ, Tuber biomass Ⅰ; TBⅡ, Tuber biomass Ⅱ; TBD, Tuber biomass decrease; TS, tundra swan; WL, Water level; WNC, white–naped crane; WT, Water table.

Four crane species preferred foraging at higher water levels (Siberian crane = 13.00 ± 0.46 m, hooded crane = 13.01 ± 0.48 m, white‐naped crane = 12.37 ± 0.74 m, common crane = 12.60 ± 0.75 m) than the three goose species. In addition, the cranes usually roosted far away from the road and foraged at the center of the sub‐lakes. Conversely, the geese roosted close to the road. The foraging habitats of tundra swans were the nearest to the village (DV = 643.00 ± 186.58 m), whereas those of common cranes (DV = 1424.69 ± 444.75 m) and greater white‐fronted geese (DV = 1258.63 ± 671.57 m) were the farthest from the village.

### 3.3 Species niches and foraging habitat selection models

The principal component analysis performed on the habitat environment characteristic variables yielded three principal components (PCs) that explained 62.53% of the total variation in the analyzed samples (Table 2). The first component, PC1, had the higher Eigenvalue (4.788) among 15 variables. The characteristics with the highest correlations were CHI (0.913), CHII (0.924), CHC (0.603), CCI (0.947), CCII (0.939), and CCD (0.617), which were all positively correlated with foraging habitat selection. The second component, PC2, with the largest Eigenvalue 2.342 among 15 variables and was positively correlated with elevation (0.746), WL (0.948), and WT (0.638). Similarly, the third component, PC3, with the largest Eigenvalue 2.250 among 15 variables and was positively correlated with TBI (0.911), TBII (0.666), and TBD (0.743).

**TABLE 2:** Principal component loading for foraging site variables

	Component		
	PC1	PC2	PC3
Eigenvalue	4.788	2.342	2.250
Percentage of variance (%)	31.918	15.611	15.001
Cumulative percentage of variance (%)	31.918	47.529	62.530
Correlation of components to Environmental factors			
TBⅠ	−0.301	−0.152	**0.911**
TBⅡ	−0.211	−0.350	**0.666**
TBD	−0.257	0.176	**0.743**
CHⅠ	**0.913**	−0.059	0.217
CHⅡ	**0.924**	−0.069	0.215
CHC	**0.603**	−0.206	0.065
CCⅠ	**0.947**	−0.071	0.160
CCⅡ	**0.939**	−0.089	0.176
CCD	**0.617**	0.152	−0.089
Elev	0.322	**0.746**	−0.064
WL	0.058	**0.948**	0.093
WT	−0.343	**0.638**	0.250
DR	0.052	0.306	0.407
DV	0.382	0.349	−0.019
DC	0.034	−0.030	−0.150

Note

Loading of variable with absolute value >0.6 are marked in bold. Variable descriptions are found in Table 1.

**FIGURE 5:**
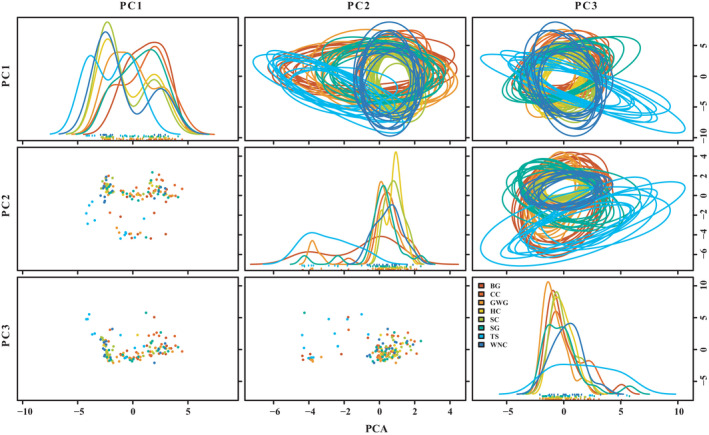
Ten random elliptical projections of trophic niche region for each bird species in Poyang lakes (elliptical plots). Also displayed are one‐dimensional density plots (lines) and two‐dimensional scatterplots. The eight waterbird species displayed are BG, bean goose; CC, common crane; GWG, greater white‐fronted goose; HC, hooded crane; SC, Siberian crane; SG, swan goose; TS, tundra swan; WNC, white‐naped crane

**FIGURE 6:**
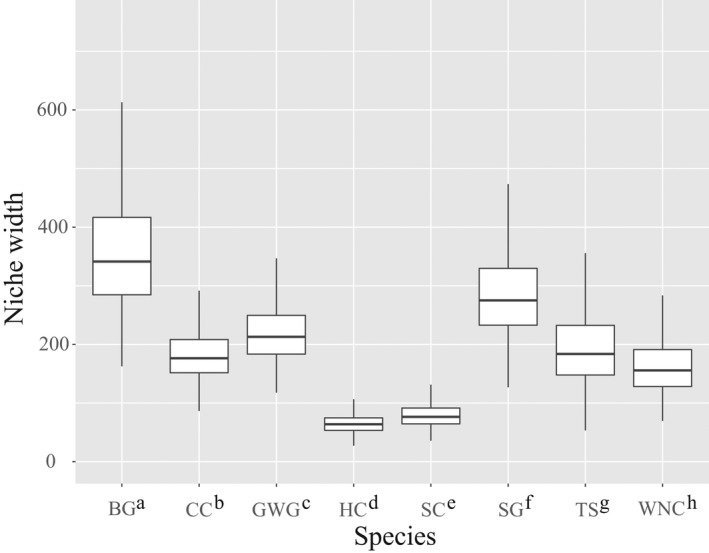
Niche width of eight waterbird species in Poyang lakes. BG, bean goose; CC, common crane; GWG, greater white‐fronted goose; HC, hooded crane; SC, Siberian crane; SG, swan goose; TS, tundra swan; WNC, white‐naped crane

Overall, all eight waterbird species showed obvious niche differentiation. The highest niche overlap probability was obtained for WNC‐SC (91.78%, Figure 5 and Table S5), whereas the lowest value was obtained from TS‐SC (0.07%). In addition, the niche overlap of TS and other birds were clearly differentiated (value < 50.00%). Our results also indicated that BG had the narrowest niche width and HC the widest niche among the niche width of the eight waterbird species (Figure 6). Niche width was significantly different among the eight waterbird species (*F* = 2508.27, df = 7, *p* < .001), and LSD test results showed all species, niches were significantly different (LSD: ALL *P* values <.001, Table S6) for eight waterbird species. We identified the best foraging habitat selection model (top‐rank model, ΔAICc = 0) for the eight waterbird species (Table 3). The results of Poisson regression indicated that the model (TBD + DV) was the top‐ranked model for the prediction of foraging habitat selection for the Siberian (*ω_i_* = 0.645) and hooded cranes (*ω_i_* = 0.630), while the model (*ω_i_* = 0.531) (TBD + DC) was the best model for the prediction of that of white‐naped cranes. The best model (*ω_i_* = 0.296) for the common crane was TBD + WL + DR + DC, where all factors except WL (*z* = −4.474, *p* = .000) had significant positive effects. The top‐ranked model (*ω_i_* = 0. 640) for the greater white‐fronted goose was CHC + CCD + DR, where all factors except CHC (*z* = −4.936, *p* = .000) had significant positive effects. Similarly, the top‐ranked model (*ω_i_* = 0.528) for the bean goose was CHC + CCD, where all factors except CHC (*z* = −7.819, *p* = .000) had significant positive effects. The best model (*ω_i_* = 0.359) for the swan geese was TBD + CCD, and all parameters in this model had significant positive effects. The best model (*ω_i_* = 0.404) for the tundra swan contained four variables (TBD + CHC + CCD + DC), where all factors except CHC (*z* = −4.936, *p* = .000) had significant positive effects (Table S7).

**TABLE 3:** Results from foraging habitat selection models of eight bird species based on corrected Akaike information criterion (AICc)

Species	Best model parameters	*k*	AICc	△AICc	*ω_i_*
SC	TBD + DV	3	76.603	0.000	0.645
HC	TBD + DV	3	162.330	0.000	0.630
WNC	TBD + DC	3	70.772	0.000	0.531
CC	TBD + WL + DR + DC	5	197.850	0.000	0.296
GWG	CHC + CCD + DR	4	212.130	0.000	0.640
BG	CHC + CCD	3	173.910	0.000	0.528
SG	TBD + CCD	3	164.950	0.000	0.359
TS	TBD + CHC + CCD + DC	5	99.817	0.000	0.404

Note

*K* is number of parameters. AICc is Akaike's information criterion corrected for small sample size. ΔAICc is difference in AICc relative to minimum AICc. *ω_i_* is the Akaike model weight. Variable descriptions are found in Table 2.

## 4 DISCUSSION

### 4.1 Species coexistence maintained by niche segregation

Niche segregation among waterbirds with respect to food types, and spatial location allow them to coexist and use varied food resources. The niche of a species as a hyper‐volume proposed by Hutchinson (1957), allows us to understand how closely related species coexist by exploiting different environmental resources. The stable coexistence of sympatric species can be attributed to ecological niche differentiation, which suggests that species may specialize in different food resources. In our study, niche overlap was observed for the eight species of wintering herbivore waterbirds at Poyang Lake (Figure 5). Based on the results of previous dietary studies, these wintering waterbird species can be divided into the following two feeding groups: *Carex* spp. foragers and tuber eaters (Barzen et al., 2009; Wang, Fox, et al., 2013; Wang, Jia, et al., 2013); this is consistent with our results from the Poisson regression models (Table 3, Table S7). In general, species with overlapping dietary niches may compete strongly if food sources are limited. Consequently, waterbirds prefer different food sources to mitigate interspecific competition, thereby allowing multiple species to forage simultaneously within the same region (Arcos et al., 2019; Henry & Cumming, 2017; Schoener, 1974).

When competing for the same food resource, two (or more) species can coexist in overlapping distribution areas via using the same resource at different time or spatial locations (Han et al., 2019; Jean‐Baptiste et al., 2012; Tokeshi, 1998). Thus, species that use the similar food resources are susceptible to competitive interactions over sharing limited resources, and to coexist, they should exert a mechanism to reduce, but not necessarily eliminate, negative competitive interactions (Chatterjee et al., 2020; Simberloff & Dayan, 1991). In this study, there was large temporal variation in waterbird abundance in surveyed areas (Figure 2). For *Carex* spp. foragers, the individual number peaked at Huangjinzui in mid‐November and around January for the other three sub‐lakes. Moreover, a small peak in waterbird numbers occurred at the three sub‐lakes between February and March. The overall trend for tuber eaters was similar to that of *Carex* spp. foragers, but the overwintering peak in Meixihu and Changhuchi was earlier than that in Baishahu. Additionally, the results of the present study suggest that direct competition among wintering waterbirds was avoided via the occupation of different WTs on a spatial scale, which helped to allocate resources more efficiently (Figure 3). For example, with regard to tuber‐eating waterbirds, the white‐naped crane, Siberian crane, and hooded crane forage in mudflats and shallow water, mainly in shallow water habitats, whereas the common crane and swan goose in grasslands and mudflats, mainly in mudflat habitats. The distribution of tundra swans was wider than that of other herbivores. Although the tuber‐eating waterbirds were distributed in a similar region of habitat, they occupied different WTs (Table 1). Thus, birds segregated their niches by occupying different WTs, thereby minimizing niche overlap. Moreover, we found that the niche width among eight waterbird species exhibited significant variation in this study (Figure 6). Based on results from PC1, PC2, and PC3, the niche width and overlap of the tundra swans exhibited marked differences with other waterbird species (Figures 5 and 6). Similarly, other foragers of the same group showed different degrees of niche separation. For example, the bean goose owned the narrowest niche, while the hooded crane had the widest niche in wintering habitats (Figure 6). Niche differences allow species to complement each other and make better use of existing resources (Büchi & Vuilleumier, 2014; Carroll et al., 2011; Northfield et al., 2010).

### 4.2 Abundance of wintering birds correlated with habitat

Habitat quality, including availability of food resources and suitable habitat area, influences the abundance and distribution of waterbird species and determine coexistence. In floodplain systems, water level fluctuations have an important influence on habitat structure and quality, for example, with regard to vegetation coverage and food availability (Clausen, 2000; Paracuellos, 2006; Wang, Fox, et al., 2013; Wang, Jia, et al., 2013). Because the water level is controlled by a sluice, the sub‐lakes of Poyang Lake have unique topographic characteristics and form special hydrological fluctuations, resulting in the spatial distribution pattern observed for the wetland vegetation. Herbivorous waterbirds used different wintering sites within one or more sub‐lakes of Poyang Lake and forage various on different wetland vegetation as food sources. Our results showed that *Carex* spp. were distributed at high elevations in the study sites, which was closely related to the water recession observed in the early overwintering period (Figure S2). In the middle and low elevation areas, the growth of *Carex* spp. was largely in response to lake water level (Feng et al., 2020; Li, Qian, et al., 2019; Li, Yu, et al., 2019). As the water level decreased, areas at high elevations were firstly exposed; this could provide a moderately environment for the growth of *Carex* spp. (Aharon‐Rotman et al., 2017; Barzen, 2008). However, it is noteworthy that the abundance of geese inhibited the growth of meadows with short *Carex* spp. (Table S3), indicating that geese preferred to forage for *Carex* spp. at the early growth stage wherein *Carex* spp. contains high protein and low structural carbohydrate content (Cadieux et al., 2005; Zhang et al., 2016). Therefore, the resource quality of vegetation suitable for *Carex* spp. foragers was strongly affected by the water level of the sub‐lakes (Hassall et al., 2001). Previous studies have suggested that floods that receded rapidly or much earlier could accelerate the exposure and growth of sedge meadows, making them unsuitable for wintering geese use (Hassall et al., 2001; Wang, Fox, et al., 2013; Wang, Jia, et al., 2013). The gradual recession in the water level of the sub‐lakes ensured sustainable access to food resources for overwintering waterbirds.

Our results suggested that water depth and the inundation duration of the wet season had significant adverse effects on the tuber biomass of submerged vegetation (Figure 4). Water depth and inundation duration can influence the distribution and growth of submerged vegetation by limiting light intensity, thereby affecting their photosynthetic ability (Li et al., 2020). Few submerged vegetation tubers could survive in Huangjinzui because the water depth was relatively high in the wet season and rapidly receded during the dry season. Therefore, the unique hydrological variation in the sub‐lakes of Poyang Lake could provide diverse foraging habitats and abundant food sources for wintering herbivore waterbirds.

Recent studies suggested that herbivorous waterbirds were restricted to winter in several better connected lakes since more lakes had been hydrological isolated from the middle and lower areas of the Yangtze River, particularly for Poyang Lake (Xia et al., 2017). Poyang Lake is a favorable wintering ground for herbivorous waterbirds because of the high production of wetland vegetation, namely, submerged macrophytes such as *Vallisneria spiralis*. However, the degradation of other wetland ecosystems is widespread in this region due to eutrophication and loss of natural hydrological connectivity (Jia et al., 2018; Wang, Gu et al., 2019; Wang, Fraser, & Chen, 2019; Xia et al., 2016), resulting in habitat loss. For example, the distribution of *Carex* spp. and tubers of *Vallisneria* spp. in Tai Lake has been lost because of eutrophication and persistent high water levels (Qin et al., 2007; Zhao et al., 2017). Similar observations were made at Shengjin Lake, precluding the availability of favorable habitats for the early arrival of wintering herbivorous waterbirds (Fox et al., 2011; Li et al., 2018). However, Poyang Lake has high wetland vegetation productivity and has become one of the most important wintering grounds for waterbirds in the East Asian‐Australasian Flyway (Barter et al., 2005; Wang, Fox, et al., 2013; Wang, Jia, et al., 2013).

### 4.3 Implication for conservation

The findings of this study demonstrated that differences in habitat use could mitigate interspecific competition, which may explain the mechanism through which waterbirds of Poyang Lake can coexist during the wintering period, despite considerable overlap in the dietary niches of herbivorous waterbirds. Therefore, we should adopt some strategies to protect waterbirds for wintering, such as protecting existing resources, and recovering of submerged vegetation and benthic invertebrates. In doing so, it could be possible to mitigate competition promoting the abundance of food resources. During the non‐breeding period, investigation of the coexistence of species and the utilization of existing resources may provide significant insights into the conservation and management of wintering waterbirds and their habitat in lakes systems. Thus, our findings may inspire more effective management of other lacustrine wetlands with similar hydrological regimes.

## CONFLICT OF INTEREST

None declared.

## AUTHOR CONTRIBUTIONS

**Junpeng Bai:** Data curation (equal); Formal analysis (equal); Methodology (equal); Visualization (equal); Writing‐original draft (equal). **Huan Zhang:** Writing‐original draft (equal); Writing‐review & editing (equal). **Hongkang Zhou:** Data curation (equal); Investigation (equal). **Shu Li:** Resources (equal); Visualization (equal). **Bin Gao:** Data curation (equal); Investigation (equal). **Peng Chen:** Data curation (equal); Investigation (equal). **Long Ma:** Investigation (equal); Visualization (equal). **Zhifeng Xu:** Data curation (equal); Investigation (equal). **Zhen Zhang:** Data curation (equal); Investigation (equal). **Changxin Xu:** Data curation (equal); Investigation (equal). **Luzhang Ruan:** Conceptualization (equal); Funding acquisition (equal); Project administration (equal); Resources (equal); Supervision (equal); Writing‐review & editing (equal). **Gang Ge:** Project administration (equal); Resources (equal).

## Supporting information

Fig S1

Fig S2

Table S1

Table S2

Table S3

Table S4

Table S5

Table S6

Table S7

## Data Availability

All data are available on Figshare (https://doi.org/10.6084/m9.figshare.14627250).
