# Supplementary figures and images for: Winter coexistence in herbivorous waterbirds: Niche differentiation in a floodplain, Poyang Lake, China
Source: Ecol Evol. 2021 Nov 15;11(23):16835–48. doi: 10.1002/ece3.8314 (PMC8668764; doi:10.1002/ece3.8314)

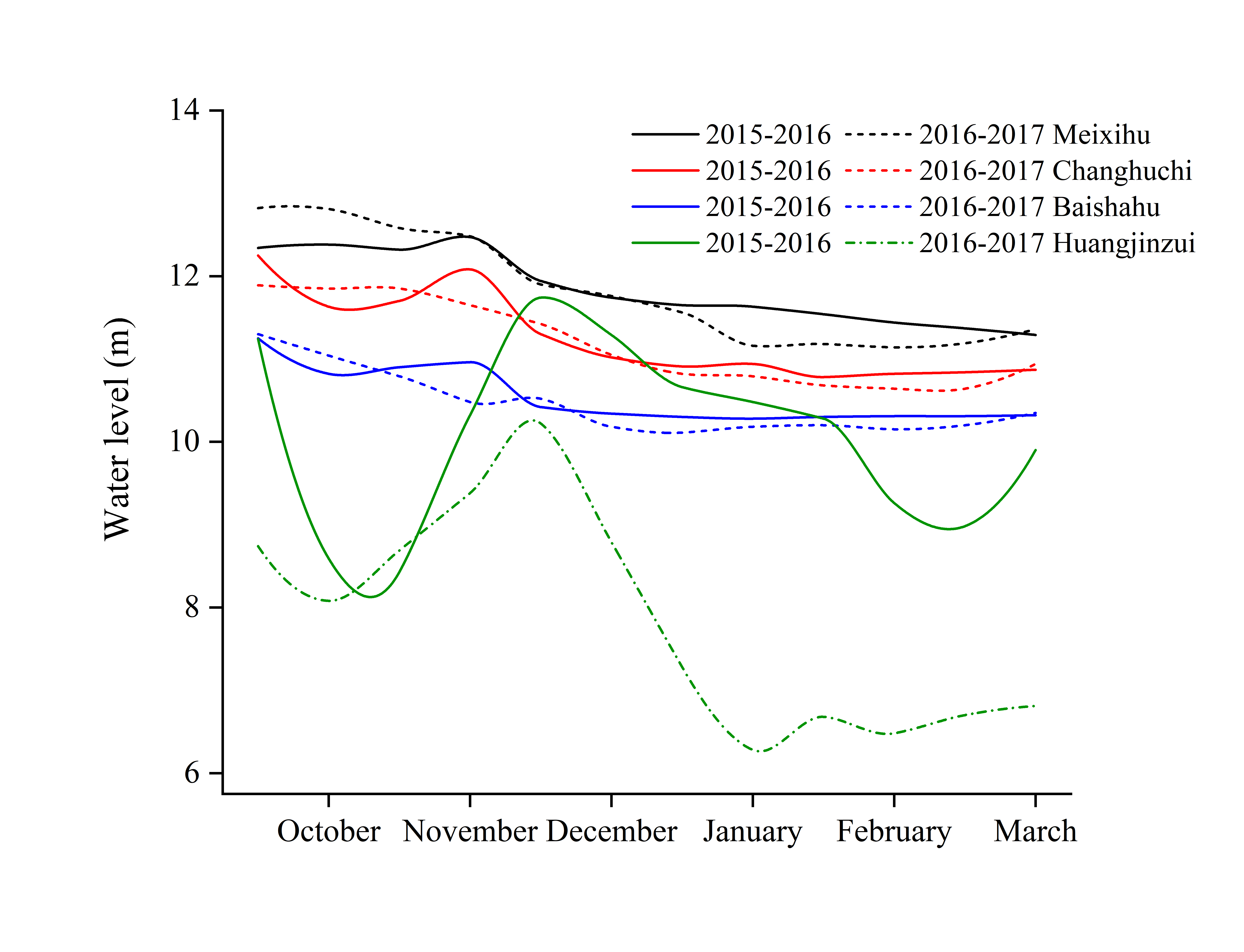

Supplement: Supplementary file 1 — Fig S1 [file ECE3-11-16835-s009.tif]

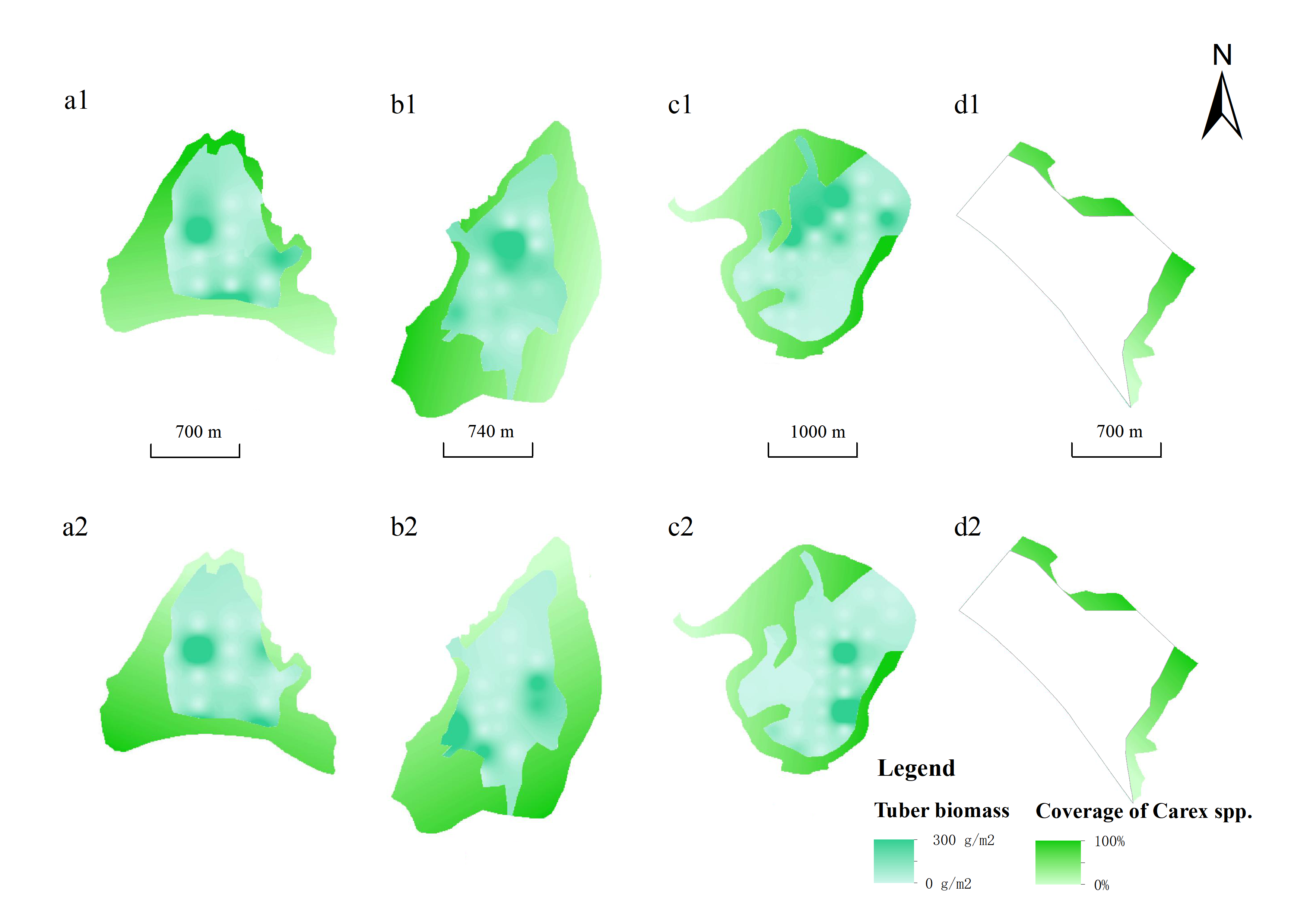

Supplement: Supplementary file 2 — Fig S2 [file ECE3-11-16835-s006.tif]
