# Supplementary material for: Winter coexistence in herbivorous waterbirds: Niche differentiation in a floodplain, Poyang Lake, China
Source: Ecol Evol. 2021 Nov 15;11(23):16835–48. doi: 10.1002/ece3.8314 (PMC8668764; doi:10.1002/ece3.8314)
Supplement: Supplementary file 3 — Table S1 [file ECE3-11-16835-s001.docx]

| Table S1 Characteristics of habitat parameters of eight wterbird species. | | | |
| --- | --- | --- | --- |
| Factors | Acronym | Description | Unit |
| Tuber biomass Ⅰ | TBⅠ | Biomass of submerged tubers in the early overwintering | g/m^2^ |
| Tuber biomass Ⅱ | TBⅡ | Biomass of submerged tubers in the late overwintering | g/m^2^ |
| Tuber biomass decrease | TBD | Decrease of biomass of submerged tubers in the early and late overwintering | g/m^2^ |
| Carex height Ⅰ | CHⅠ | Height of *Carex* pp. in the early overwintering | cm |
| Carex height Ⅱ | CHⅡ | Height of *Carex* pp. in the late overwintering | cm |
| Carex height changes | CHC | Change of *Carex* pp. height in the early and late overwintering | cm |
| Carex coverage Ⅰ | CCⅠ | Coverage of *Carex* pp. in the early overwintering | % |
| Carex coverageⅡ | CCⅡ | Coverage of *Carex* pp. in the late overwintering | % |
| Carex coverage decrease | CCD | Change of *Carex* pp. coverage in the early and late overwintering | % |
| Elevation | Elev | Elevation of the land above the base of the Yellow Sea Datum | m |
| Water level | WL | The free surface of water body is elevated above the base plane of the Yellow Sea Datum | m |
| Water Table | WT | The difference between elevation and water level | cm |
| Distance from road | DR | Distance to the nearest road as the crow flies | m |
| Distance from village | DV | Distance to the nearest village as the crow flies | m |
| Distance from center | DC | Distance to the nearest center of the lake | m |
