# Supplementary material for: Winter coexistence in herbivorous waterbirds: Niche differentiation in a floodplain, Poyang Lake, China
Source: Ecol Evol. 2021 Nov 15;11(23):16835–48. doi: 10.1002/ece3.8314 (PMC8668764; doi:10.1002/ece3.8314)
Supplement: Supplementary file 4 — Table S2 [file ECE3-11-16835-s003.docx]

| Table S2 Kolmogorov–Smirnov test of habitat characteristics. | | | |
| --- | --- | --- | --- |
|  | *D* value | *P* value | Distribution |
| TBⅠ | 3.084 | 0.000 | abnormal |
| TBⅡ | 4.658 | 0.000 | abnormal |
| TBD | 2.801 | 0.000 | abnormal |
| CHⅠ | 2.386 | 0.000 | abnormal |
| CHⅡ | 2.418 | 0.000 | abnormal |
| CHC | 2.528 | 0.000 | abnormal |
| CCⅠ | 2.484 | 0.000 | abnormal |
| CCⅡ | 2.425 | 0.000 | abnormal |
| CCD | 2.464 | 0.000 | abnormal |
| Elev | 2.196 | 0.000 | abnormal |
| WL | 3.593 | 0.000 | abnormal |
| WT | 2.727 | 0.000 | abnormal |
| DR | 1.099 | 0.178 | normal |
| DV | 1.159 | 0.136 | normal |
| DC | 2.703 | 0.000 | abnormal |
| TBⅠ= Tuber biomass Ⅰ, TBⅡ= Tuber biomass Ⅱ, TBD = Tuber biomass decrease, CHⅠ = *Carex* spp. height Ⅰ, CHⅡ = *Carex* spp. height Ⅱ, CHC = *Carex* spp. height changes, CCⅠ = *Carex* spp. coverage Ⅰ, CCⅡ = *Carex* spp. coverage Ⅱ, CCD = *Carex* spp. coverage decrease, Elev = Elevation, WL = Water level, WT = Water table, DR = Distance from road, DV = Distance from village, DC = Distance from center. | | | |
