# Supplementary material for: Winter coexistence in herbivorous waterbirds: Niche differentiation in a floodplain, Poyang Lake, China
Source: Ecol Evol. 2021 Nov 15;11(23):16835–48. doi: 10.1002/ece3.8314 (PMC8668764; doi:10.1002/ece3.8314)
Supplement: Supplementary file 5 — Table S3 [file ECE3-11-16835-s002.docx]

| Table S3 Spearman's rank correlation test (r) of habitat characteristics. | | | | | | | | | | | | | | | |
| --- | --- | --- | --- | --- | --- | --- | --- | --- | --- | --- | --- | --- | --- | --- | --- |
|  | TBI | TBII | TBD | CHI | CHII | CHC | CCI | CCII | CCD | Elev | WL | WT | DR | DV | DC |
| TBI | 1 |  |  |  |  |  |  |  |  |  |  |  |  |  |  |
| TBII | 0.763^**^ | 1 |  |  |  |  |  |  |  |  |  |  |  |  |  |
| TBD | 0.958^**^ | 0.603^**^ | 1 |  |  |  |  |  |  |  |  |  |  |  |  |
| CHI | -0.142 | -0.129 | -0.128 | 1 |  |  |  |  |  |  |  |  |  |  |  |
| CHII | -0.142 | -0.117 | -0.132 | 0.998^**^ | 1 |  |  |  |  |  |  |  |  |  |  |
| CHC | -0.181^*^ | -0.034 | -0.224^**^ | 0.517^**^ | 0.541^**^ | 1 |  |  |  |  |  |  |  |  |  |
| CCI | -0.187^*^ | -0.197^*^ | -0.171^*^ | 0.910^**^ | 0.913^**^ | 0.552^**^ | 1 |  |  |  |  |  |  |  |  |
| CCII | -0.188^*^ | -0.197* | -0.175^*^ | 0.914^**^ | 0.917^**^ | 0.570^**^ | 0.998^**^ | 1 |  |  |  |  |  |  |  |
| CCD | -0.188^*^ | -0.174^*^ | -0.163^*^ | 0.635^**^ | 0.628^**^ | 0.184^*^ | 0.687^**^ | 0.649^**^ | 1 |  |  |  |  |  |  |
| Elev | 0.146 | -0.025 | 0.251^**^ | 0.262^**^ | 0.253^**^ | -0.036 | 0.168^*^ | 0.163^*^ | 0.229^**^ | 1 |  |  |  |  |  |
| WL | 0.253^**^ | 0.019 | 0.361^**^ | 0.007 | -0.008 | -0.228^**^ | -0.081 | -0.088 | 0.043 | 0.896^**^ | 1 |  |  |  |  |
| WT | 0.292^**^ | 0.127 | 0.319^**^ | -.681^**^ | -0.686^**^ | -0.479^**^ | -0.632^**^ | -.643^**^ | -0.401^**^ | 0.017 | 0.358^**^ | 1 |  |  |  |
| DR | 0.412^**^ | 0.269^**^ | 0.448^**^ | 0.020 | 0.031 | 0.089 | 0.058 | 0.062 | -0.107 | 0.125 | 0.166^*^ | 0.121 | 1 |  |  |
| DV | -0.116 | -0.139 | -0.057 | 0.190^*^ | 0.207^**^ | 0.261^**^ | 0.293^**^ | 0.291^**^ | 0.149 | 0.128 | 0.083 | 0.050 | 0.351^**^ | 1 |  |
| DC | -0.097 | -0.032 | -0.137 | 0.151 | 0.141 | -0.086 | 0.197^*^ | 0.183^*^ | 0.351^**^ | -0.072 | -0.114 | -0.149 | -0.333^**^ | -0.151 | 1 |
| ** Significant correlation at the 0.01 level. * Significant correlation at the 0.05 level. TBI= Tuber biomass I, TBII= Tuber biomass II, TBD = Tuber biomass decrease, CHI = *Carex* height I, CHII = *Carex* height II, CHC = *Carex* height changes, CCI = *Carex* coverage I, CCII = *Carex* coverage II, CCD = *Carex* coverage decrease, Elev = Elevation, WL = Water level, WT = Water table, DR = Distance from road, DV = Distance from village, DC = Distance from center. | | | | | | | | | | | | | | | |
