# Supplementary material for: Winter coexistence in herbivorous waterbirds: Niche differentiation in a floodplain, Poyang Lake, China
Source: Ecol Evol. 2021 Nov 15;11(23):16835–48. doi: 10.1002/ece3.8314 (PMC8668764; doi:10.1002/ece3.8314)
Supplement: Supplementary file 6 — Table S4 [file ECE3-11-16835-s008.docx]

| Table S4 Number of wintering waterbirds in four study areas of Poyang Lake between 2015/2016 and 2016/2017. | | | | |
| --- | --- | --- | --- | --- |
| Year | Month | Carex spp. foragers | Tuber eaters | All |
| 2015/2016 |  |  |  |  |
|  | October | 134 | 35 | 169 |
|  | November | 9033 | 1306 | 10339 |
|  | December | 9820 | 2803 | 12623 |
|  | January | 14337 | 9540 | 23877 |
|  | February | 12373 | 4352 | 16725 |
|  | March | 9872 | 5966 | 15838 |
| 2016/2017 |  |  |  |  |
|  | October | 221 | 85 | 306 |
|  | November | 13109 | 1500 | 14609 |
|  | December | 13223 | 11843 | 25066 |
|  | January | 10383 | 9988 | 20371 |
|  | February | 1900 | 935 | 2835 |
|  | March | 3066 | 3279 | 6345 |
|  | Mean | 8122 | 4302 | 12425 |
