# Supplementary material for: Winter coexistence in herbivorous waterbirds: Niche differentiation in a floodplain, Poyang Lake, China
Source: Ecol Evol. 2021 Nov 15;11(23):16835–48. doi: 10.1002/ece3.8314 (PMC8668764; doi:10.1002/ece3.8314)
Supplement: Supplementary file 7 — Table S5 [file ECE3-11-16835-s004.docx]

| Species | BG | CC | GWG | HC | SC | SG | TS | WNC |
| --- | --- | --- | --- | --- | --- | --- | --- | --- |
| BG |  | 44.83 | 65.61 | 18.75 | 18.77 | 68.25 | 17.72 | 36.08 |
| CC | 82.92 |  | 74.36 | 56.35 | 56.69 | 87.1 | 1.71 | 77.44 |
| GWG | 83.41 | 63.29 |  | 36.09 | 38.56 | 78.26 | 9.21 | 59.74 |
| HC | 77.67 | 91.30 | 90.23 |  | 84.54 | 88.73 | 0.40 | 89.46 |
| SC | 69.15 | 87.23 | 83.74 | 75.94 |  | 78.74 | 0.45 | 91.78 |
| SG | 81.54 | 68.08 | 65.39 | 33.5 | 33.83 |  | 6.07 | 54.35 |
| TS | 33.15 | 1.53 | 11.23 | 0.07 | 0.09 | 15.29 |  | 1.31 |
| WNC | 63.18 | 80.48 | 69.8 | 54.12 | 63.08 | 71.45 | 2.53 |  |

Table S5 The pairwise of niche overlap probability for 8 waterbird species in Poyang Lake.
