# Supplementary material for: Winter coexistence in herbivorous waterbirds: Niche differentiation in a floodplain, Poyang Lake, China
Source: Ecol Evol. 2021 Nov 15;11(23):16835–48. doi: 10.1002/ece3.8314 (PMC8668764; doi:10.1002/ece3.8314)
Supplement: Supplementary file 8 — Table S6 [file ECE3-11-16835-s007.docx]

| Species | BG | SG | GWG | TS | CC | WNC | SC | HC |
| --- | --- | --- | --- | --- | --- | --- | --- | --- |
| BG |  | *** | *** | *** | *** | *** | *** | *** |
| SG | 69.53 |  | *** | *** | *** | *** | *** | *** |
| GWG | 138.19 | 68.66 |  | *** | *** | *** | *** | *** |
| TS | 158.12 | 88.60 | 19.94 |  | *** | *** | *** | *** |
| CC | 175.90 | 106.38 | 37.72 | 17.78 |  | *** | *** | *** |
| WNC | 194.80 | 125.28 | 56.62 | 36.68 | 18.90 |  | *** | *** |
| SC | 278.97 | 209.44 | 140.78 | 120.84 | 103.06 | 84.16 |  | *** |
| HC | 292.93 | 223.40 | 154.75 | 134.81 | 117.03 | 98.13 | 13.97 |  |

TableS6 The pairwise comparison of niche width for eight waterbird species in Poyang lakes. The left bottom represents the mean differences in niche widths of pairwise comparison species, the top right corner indicates a significant difference, and significant level of *p* value at 0.001 were shown as ***.
