# Supplementary material for: Winter coexistence in herbivorous waterbirds: Niche differentiation in a floodplain, Poyang Lake, China
Source: Ecol Evol. 2021 Nov 15;11(23):16835–48. doi: 10.1002/ece3.8314 (PMC8668764; doi:10.1002/ece3.8314)
Supplement: Supplementary file 9 — Table S7 [file ECE3-11-16835-s005.docx]

| Table S7 Significance test of foraging site selection best model regression coefficient. | | | | | |
| --- | --- | --- | --- | --- | --- |
| Species | Parameters | Coefficients | SE | z | *P* |
| SC | Intercept | 0.052 | 0.273 | 0.189 | 0.850 |
|  | TBD | 0.015 | 0.003 | 5.423 | **0.000** |
|  | DV | 0.001 | 0.000 | 3.768 | **0.000** |
| HC | Intercept | 1.489 | 0.136 | 10.966 | **0.000** |
|  | TBD | 0.015 | 0.001 | 15.858 | **0.000** |
|  | DV | 0.001 | 0.000 | 6.621 | **0.000** |
| WNC | Intercept | -0.079 | 0.293 | -0.269 | 0.788 |
|  | TBD | 0.015 | 0.003 | 5.822 | **0.000** |
|  | DC | 0.001 | 0.000 | 2.884 | **0.004** |
| CC | Intercept | 5.928 | 1.226 | 4.834 | **0.000** |
|  | TBD | 0.010 | 0.002 | 5.913 | **0.000** |
|  | WL | -0.485 | 0.108 | -4.474 | **0.000** |
|  | DR | 0.001 | 0.000 | 2.710 | **0.007** |
|  | DC | 0.002 | 0.000 | 5.204 | **0.000** |
| GWG | Intercept | 2.055 | 0.114 | 18.101 | **0.000** |
|  | CHC | -0.557 | 0.113 | -4.936 | **0.000** |
|  | CCD | 11.065 | 1.876 | 5.897 | **0.000** |
|  | DR | 0.001 | 0.000 | 4.016 | **0.000** |
| BG | Intercept | 3.238 | 0.116 | 27.824 | **0.000** |
|  | CHC | -1.919 | 0.245 | -7.819 | **0.000** |
|  | CCD | 19.144 | 2.446 | 7.826 | **0.000** |
| SG | Intercept | 1.806 | 0.129 | 13.958 | **0.000** |
|  | TBD | 0.015 | 0.002 | 8.467 | **0.000** |
|  | CCD | 19.535 | 1.820 | 10.732 | **0.000** |
| TS | Intercept | -0.522 | 0.931 | -0.560 | 0.575 |
|  | TBD | 0.010 | 0.002 | 4.211 | **0.000** |
|  | CHC | 1.952 | 0.868 | 2.248 | **0.025** |
|  | CCD | -44.140 | 13.230 | -3.337 | **0.001** |
|  | DC | 0.003 | 0.001 | 3.122 | **0.002** |
| SE: standard error. Statistically significant effects are shown in bold. Variable descriptions are found in Table 1. | | | | | |
